# p16^INK4a^ Regulates Cellular Senescence in PD-1-Expressing Human T Cells

**DOI:** 10.3389/fimmu.2021.698565

**Published:** 2021-08-09

**Authors:** Valérie Janelle, Mathieu Neault, Marie-Ève Lebel, Dave Maurice De Sousa, Salix Boulet, Ludovic Durrieu, Cédric Carli, Chloé Muzac, Sébastien Lemieux, Nathalie Labrecque, Heather J. Melichar, Frédérick A. Mallette, Jean-Sébastien Delisle

**Affiliations:** ^1^Research Centre, Hôpital Maisonneuve-Rosemont, Montreal, QC, Canada; ^2^Department of Microbiology, Infectious Diseases and Immunology, Université de Montréal, Montreal, QC, Canada; ^3^Institute for Research in Immunology and Cancer, Université de Montréal, Montreal, QC, Canada; ^4^Department of Biochemistry and Molecular Medicine, Université de Montréal, Montreal, QC, Canada; ^5^Department of Medicine, Université de Montréal, Montreal, QC, Canada; ^6^Division of Hematology-Oncology, Hôpital Maisonneuve-Rosemont, Montreal, QC, Canada

**Keywords:** T cells, exhaustion and activation markers, cellular senescence, p38MAPK, p16^INK4a^, adoptive immunotherapy, CAR T cells

## Abstract

T-cell dysfunction arising upon repeated antigen exposure prevents effective immunity and immunotherapy. Using various clinically and physiologically relevant systems, we show that a prominent feature of PD-1-expressing exhausted T cells is the development of cellular senescence features both *in vivo* and *ex vivo*. This is associated with p16^INK4a^ expression and an impaired cell cycle G1 to S-phase transition in repeatedly stimulated T cells. We show that these T cells accumulate DNA damage and activate the p38MAPK signaling pathway, which preferentially leads to p16^INK4a^ upregulation. However, in highly dysfunctional T cells, p38MAPK inhibition does not restore functionality despite attenuating senescence features. In contrast, p16^INK4a^ targeting can improve T-cell functionality in exhausted CAR T cells. Collectively, this work provides insights into the development of T-cell dysfunction and identifies T-cell senescence as a potential target in immunotherapy.

**Graphical Abstract:**
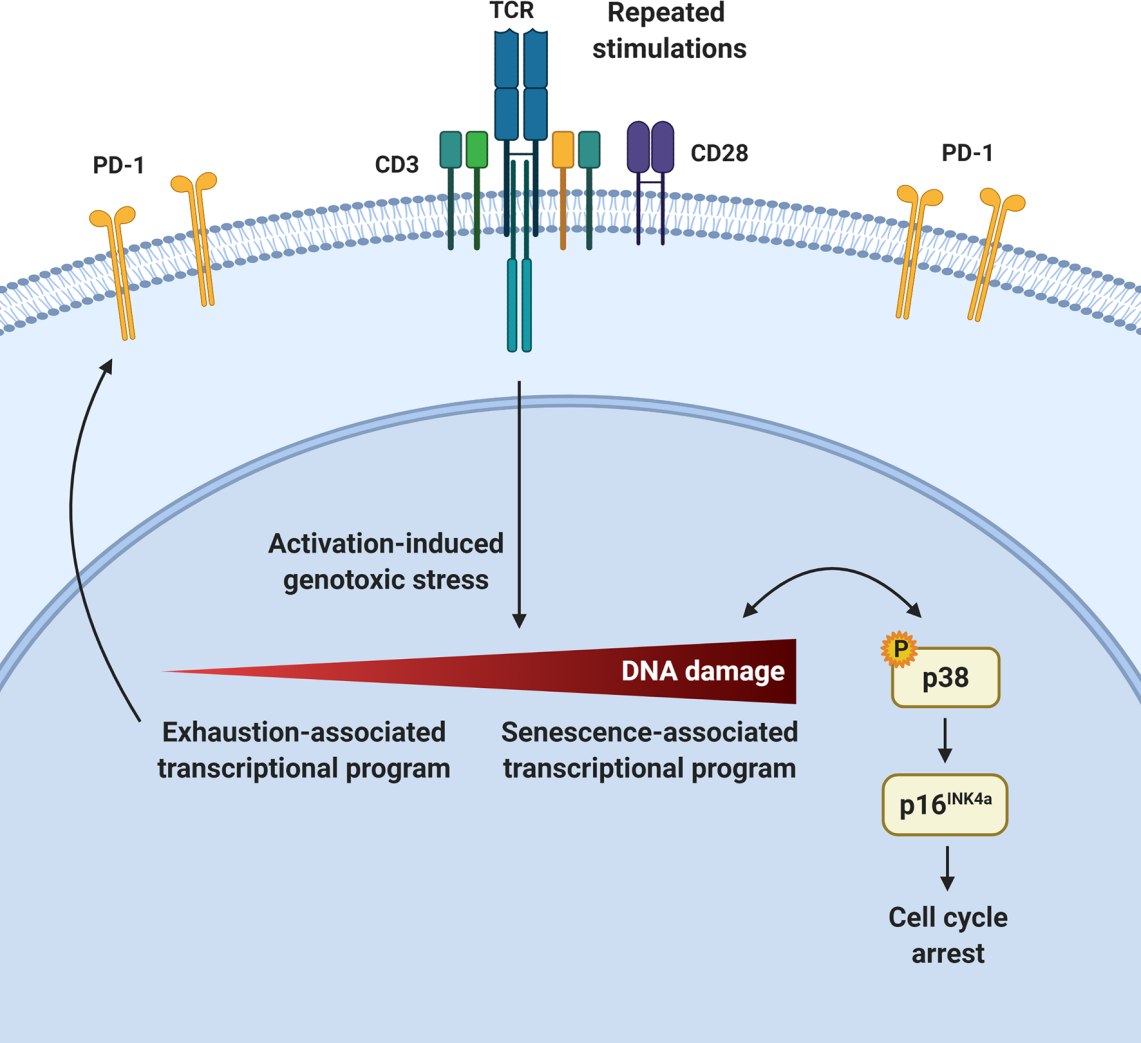


## Introduction

The capacity of conventional T cells to control or eradicate pathogens and transformed cells is compromised following repeated stimulations, owing to the development of T-cell dysfunction. The cornerstone of most current immunotherapies is to use the remarkable potential of T cells by either interfering with processes associated with T-cell dysfunction *in vivo* or manipulating T cells *ex vivo*. According to the prevailing model, T-cell differentiation is dictated by the strength and duration of T-cell stimulation (1). As such, repeated antigenic encounters in the setting of chronic infections or cancer, as well as the extensive culture required for the generation of sufficient cell numbers for treatment in certain adoptive cell therapy (ACT) settings, are conducive to the induction of T-cell terminal differentiation, exhaustion, senescence or apoptosis (2). Whether these cellular states are distinct, partially overlapping, co-regulated or part of a continuum remains incompletely understood. Moreover, several gradations within these categories exist as exemplified by variable responses of dysfunctional T cells following the blockade of the exhaustion-associated immune checkpoint PD-1 in cancer therapy (3, 4).

Senescent immune cells accumulate with organismal aging (5). Cellular senescence can be triggered by various forms of cellular stress and is characterized by prolonged cell cycle arrest as well the acquisition of several features including morphological changes, chromatin remodeling, metabolic reprogramming, and secretion of multiple factors collectively called the senescence-associated secretory phenotype (SASP) (6). The most extensively studied pathways controlling cellular senescence triggered by DNA damage involve p53/p21^Cip1^ and/or p16^INK4a^/retinoblastoma (Rb) regulators (7–10). While the p53/p21^Cip1^ pathway seems to be important at the initiation of the senescence process, p16^INK4a^ is, in addition, associated with the maintenance of the cell cycle arrest associated to senescence (11, 12). Indeed, it has been suggested that the p53/p21^Cip1^-mediated cell cycle arrest is temporary in cases of low to moderate amounts of DNA damage (13–15). However, a prolonged arrest may lead to the upregulation of the cyclin-dependant kinase inhibitor p16^INK4a^, which will activate the transcriptional regulator Rb, resulting in a permanent cell cycle arrest in G1 phase (16, 17).

The p38 mitogen-activated protein kinase (MAPK) pathway is also actively linked to the establishment of cellular senescence (18–21). Genotoxic stress leading to histone modifications and the recruitment of DNA damage sensors can trigger p38 phosphorylation and mediate its nuclear translocation (22–24). Then, activated p38 can arrest cell division by activating p53 or by directly enhancing the activity of molecules such as p16^INK4a^ to block cell cycling at the G2/M or G1/S checkpoints, respectively (20, 25, 26). A form of DNA damage-associated senescence has also been linked to p38MAPK signaling in both circulating T cells and antigen-experienced tumor infiltrating lymphocytes (TILs) (27, 28). However, the mechanisms downstream of T-cell activation and p38MAPK activation leading to cellular senescence are still elusive. Here, we show that p16^INK4a^ expression associated with cellular senescence is a cardinal feature of PD-1-expressing T cells following repeated T-cell activation in multiple experimental and clinically relevant settings and as such, represents a target to reverse T-cell dysfunction in immunotherapy.

## Materials and Methods

### Study Approval

This study was approved by the local Hôpital Maisonneuve-Rosemont research ethics authorities and participants’ informed consent was obtained (CÉR2020-2141, HQ2017-004 and CÉR13030). All animal protocols (2016-OC-024 and 2017-AV-010) were likewise approved by the local Animal Care Committee in accordance with the Canadian Council on Animal Care guidelines.

### Donors

Peripheral blood mononuclear cells (PBMCs) of healthy donors with the HLA-A0201 allele (for antigen-specific experiments) or various alleles (for anti-CD3/CD28 activation experiments) were obtained after informed consent by venipuncture, apheresis or leukoreduction system chambers (Héma-Québec) (29) followed by manual (Ficoll-Paque, GE Healthcare, Canada) or automated (Sepax system, Biosafe America Inc., Houston, TX) gradient density separation.

### Virus and Cells

BCMA-expressing human multiple myeloma KMS-11 cells (kind gift from Jonathan Bramson, McMaster University) were cultured in RPMI media (Gibco), supplemented with 10% FBS, 2 mmol/L L-glutamine (Sigma-Aldrich), penicillin (100 U/mL)/streptomycin (100 mg/mL; Sigma-Aldrich) and puromycin (InvivoGen). MC57G and L929 fibroblasts were cultured in MEM containing 5% heat-inactivated fetal bovine serum. LCMV clone 13 and Armstrong (kind gift from Alain Lamarre, Institut Armand-Frappier, Laval, Canada) were propagated by infection of the L929 fibroblast cell line and virus was harvested in the supernatant as described before (30).

### Mouse

C57BL/6 mice were purchased from The Jackson Laboratory. Mice were maintained in a specific pathogen-free environment at the Hôpital Maisonneuve-Rosemont Research Center. Female 6-12 week-old mice were infected with 2 x 10^5^ FFU (Focus Forming Units) of LCMV Armstrong intraperitoneally (i.p.) (acute infection) or 2 x 10^6^ FFU of LCMV clone 13 intravenously (i.v.) (chronic infection). The spleens were harvested 8 or 30 days post-infection for analysis.

### T-Cell Line Generation

For polyclonal activation, human T cells were enriched from PBMCs using the EasySep™ Human T Cell Enrichment Kit (StemCell) and pulsed at a 1:5 ratio (beads:cells) with anti-CD3/CD28 magnetic beads (Gibco™ Dynabeads™ Human T-Activator). Cells were cultured in G-Rex bioreactors (Wilson Wolf Manufacturing) in T-cell medium (Advanced RPMI 1640, 10% human serum, 1X L-glutamine). Cell number and viability was evaluated with a Typan Blue exclusion assay and live cells concentration was adjusted to 0.5 x 10^6^ cells/mL and restimulated weekly with beads.

Antigen-specific T cell lines were generated using 5 x 10^6^ PBMCs as responder cells and co-cultured with autologous, peptide-loaded mature dendritic cells (DCs) as antigen presenting cells (APCs) at a 1:10 ratio (stimulator:effector). DCs were generated from PBMCs that were isolated by plastic adherence and cultured in DC medium (X-vivo 15, 5% human serum, 1X PSG, 1mM sodium pyruvate) supplemented with 800IU/mL GM-CSF (Miltenyi Biotech, Germany) and 1000IU/mL IL-4 (Miltenyi Biotech). Dendritic cells were matured with GM-CSF, IL-4, TNFα (10ng/mL), IL-1β (10ng/mL), IL-6 (100ng/mL) (Miltenyi Biotech) and prostaglandin E2 (1µg/mL) (Sigma-Aldrich). After 40 Gy irradiation, the DCs were loaded with 1µg/mL LMP2_426-434_ (CLGGLLTMV) (Anaspec). Cells were cocultured for 7 days in T-cell medium (Advanced RPMI 1640, 10% human serum, 1X L-glutamine) supplemented with IL-21 (30ng/mL) and IL-12 (10ng/mL) (Miltenyi Biotech) in G-Rex 24 Well Plates (Wilson Wolf Manufacturing, New Brighton, MN). At day 7, T cells were washed and restimulated with peptide-pulsed DCs and incubated in T-cell medium supplemented with IL-21, IL-2 (100IU/mL), IL-7 (10ng/mL) and IL-15 (5ng/mL) (Miltenyi Biotech) for an additional week. Re-stimulations of T cells were performed on day 14 and 21 in T-cell medium supplemented with IL-2, IL-7 and IL-15. Cytokines were replenished with half media changes on days 11, 18, and 25. The cell concentration was adjusted to 0.5 x 10^6^ cells/mL each week.

### RNA Sequencing

Human CD8^+^ T cells activated with anti-CD3/CD28 beads in culture were enriched using the EasySep™ Human CD8^+^ T Cell Enrichment Kit (StemCell). RNA was extracted with TRIzol from unstimulated (Day 0), activated (Day 7) and dysfunctional (Day 35-49) CD8^+^ T cells. Transcriptome analysis was performed using an Illumina HiSeq 2000, TruSeq libraries, approximately 100 million reads per samples and TruSeq Small RNA library, approximately 33 million reads per sample. The data were then analyzed using Bioconductor packages (http://www.bioconductor.org/) and R statistical language (www.r-project.org). Differentially expressed genes analysis was performed using the DESeq2 package version 1.6.3 with raw read counts from AmpliSeq. Read count normalization was performed using the regularized logarithm (rlog) method provided in DESeq2. The data can be found in the Gene Expression Omnibus (GSE132727). Gene expression data from public repository of circulating CD4^+^ and CD8^+^ T cells from healthy donors or acute myeloid leukemia (AML) patients, obtained from Affymetrix Human Genome U133 Plus 2.0 Array platform [GSE14924 (31)] was also used.

Spleens from LCMV clone 13 infected mice were harvested at day 8 and 30 post-infection and mechanically dissociated. CD8^+^CD44^+^Tet-gp33^+^ cells were sorted and RNA was extracted with TRIzol reagent then further purified using RNeasy columns (Qiagen). Library preparation was done with the KAPA mRNAseq stranded library preparation kit (Roche, after mRNA capture with Dynabeads^®^ mRNA DIRECTTM Purification Kit). The data can be found in the Gene Expression Omnibus (GSE132989).

### Gene-Set Enrichment Analysis of RNA-Seq Data

Human gene symbols were ranked by the fold changes of the gene expression as profiled by RNA-seq. Then, gene-set enrichment was analyzed using GSEA 3.0 software (http://software.broadinstitute.org/gsea/downloads.jsp) (32). GSEA enrichment table files were loaded in the Enrichment Map plugin from Cytoscape software v3.2.1 (33) and filtered for significance according to the p-value (0.001) and FDR thresholds (0.01). Overlap between significant gene-sets was computed according to the Jaccard+overlap combined coefficient.

### Quantitative PCR

Cells were recovered after sorting on a FACS Aria III instrument (BD Biosciences). RNA extraction was performed by a two-step approach using Trizol (Invitrogen) and the RNeasy micro kit (Qiagen) according to the manufacturer’s instructions. After DNAse treatment (Ambion, Life technologies), RNA was reverse transcribed with random primers using the High Capacity cDNA Reverse Transcription Kit (Life Technologies) as described by the manufacturer. Real time qPCR reactions were performed using TaqMan Advanced Fast Universal PCR Master Mix (Life Technologies). The Viia7 qPCR instrument (Life Technologies) was used to detect amplification levels. Relative expression (RQ = 2^-ΔΔCT^) was calculated using the Expression Suite software (Life Technologies), and normalization was done using *HPRT*, *GAPDH* and *ACTB*.

### Telomere Measurement

Telomeres were measured by quantitative PCR as described before (34). Briefly, genomic DNA was isolated with the DNeasy kit (Qiagen). For telomere amplification (T Primer pair), the sequences are (written 5′→3′):

tel 1: CGG TTT GTT TGG GTT TGG GTT TGG GTT TGG GTT TGG GTT;
tel 2: GGC TGG CCT TAC CCT TAC CCT TAC CCT TAC CCT TAC CCT.

For the gene control 36B4 (single copy gene) amplification (S primer pair), the sequences are:

36B4u: CAG CAA GTG GGA AGG TGT AAT CC;
36B4d: CCC ATT CTA TCA TCA ACG GGT ACA A.

Relative telomere/single copy gene (T/S) ratios reflect relative length differences in telomeric DNA.

### Flow Cytometry

The phenotype of T cells was assessed at different time points of the culture by flow cytometry. To determine antigen specificity, HLA-A0201/LMP2_426-434_ dextramer (Immudex) or H2-Db/gp33 tetramer staining was performed (the H2-Db/gp33-41 biotinylated monomers were obtained through the NIH Tetramer Core Facility, and tetramers were generated using ExtrAvidin®-PE (Thermo Fisher Scientific). Cell viability was assessed by Zombie Aqua fixable viability dye. Cells were surface stained with human monoclonal antibodies against: CD3, CD4, CD8, CD45RO, CD45RA, CD62L, CCR7, PD-1, TIM3, 2B4, CD38, CD27, CD28, NGFR (BioLegend) or mouse monoclonal antibodies against: CD4, CD8, CD62L, CD69, CD44, PD-1, (BD Biosciences), before acquisition on a LSRII or FortessaX-20 instrument (BD Biosciences). For SA-β-Gal evaluation, cells were stained with the Quantitative Cellular Senescence Assay kit (Cell Biolabs). The Vybrant DyeCycle Green Stain (ThermoFisher) was used for cell cycle analysis. For T-cell function, cells were incubated with 7.5µg/mL brefeldin A (Sigma-Aldrich) for 4 hours in the presence or not of phorbol 12-myristate 13-acetate (PMA; 50ng/ml) and ionomycin (500ng/ml) (Sigma-Aldrich). Cells were permeabilized with Foxp3 staining buffer set (eBiosciences) before intracellular staining with antibodies against IFNγ and TNFα (BioLegend). For DNA damage and proliferation determination, cells were also permeabilized with Foxp3 staining buffer set prior to intracellular staining with anti-γH2AX and anti-Ki67 antibodies, respectively (BioLegend). Data were analyzed using FlowJo software (BD Biosciences).

### shRNA

The MSCV/LTRmiR30-Puro-IRES-mCherry (LMP-mCherry) empty vector was generated by replacing the GFP cassette from LMP (35) with mCherry (Addgene #52109) using *Nco*I and *Sal*I restriction sites. LMP-mCherry-shRen.713 was generated by subcloning the miR30 context *Renilla reniformis* (renilla) luciferase-targeting shRNA fragment (36) into LMP-mCherry digested with *Xho*I and *Eco*RI. LMP-mCherry-shp16 vector was generated by ligating the *Xho*I-*Eco*RI fragment from MSCV-shp16 (37) containing the U6 promoter/p16 hairpin cassette fragment into LMP-mCherry digested with *Xho*I and *Eco*RI. LMP-mCherry-shp53 was generated by ligating the *Xho*I-*Eco*RI fragment from pRetroSuper-shp53 (38) into LMP-mCherry digested with *Xho*I and *Eco*RI.

Weekly anti-CD3/CD28 stimulated T cells were transduced with retroviruses for shRNA delivery 24 hours after the third stimulation and subsequently stimulated as described above. For the transduction, cells were resuspended in viral supernatant supplemented with 10% fetal bovine serum, transferred in retronectin-coated plates (Takara) and spinoculated 90 minutes at 2250 rpm at 20°C.

### Chimeric Antigen Receptor (CAR) T-Cell Generation

Second generation B-cell maturation antigen (BCMA)-specific CAR construct [on the CD28ζ-CAR backbone and expressing the NGFR marker as described in (39, 40)] is a kind gift from Jonathan Bramson (McMaster University, Hamilton, Canada). Lentiviral transduction of T cells was performed 24 hours after stimulation with CD3/CD28-coated beads. Cells were restimulated at day 7 and 14 with beads. Cells were then transduced with shRNA-containing retroviruses as described above and co-cultured weekly with human BCMA-expressing KMS-11 cells (also a kind gift from J. Bramson) until day 35.

### Statistical Analysis

Statistical analyses were performed with R statistical language or GraphPad Prism v8.3.0. Statistical details of experiments, including number of independent donors, statistical test (depending if normal distribution was assumed according to Shapiro-Wilk testing) and statistical significance (*p*-value) are reported in the figure legends. Samples were paired when indicated. For gene expression, statistics were calculated with DESeq2 and adjusted *p*-values were used. *P-*values of less than 0.05 were considered significant.

## Results

### Repeated *Ex Vivo* Stimulations Recapitulate Phenotypic and Functional T-Cell Exhaustion

**Figure 1:**
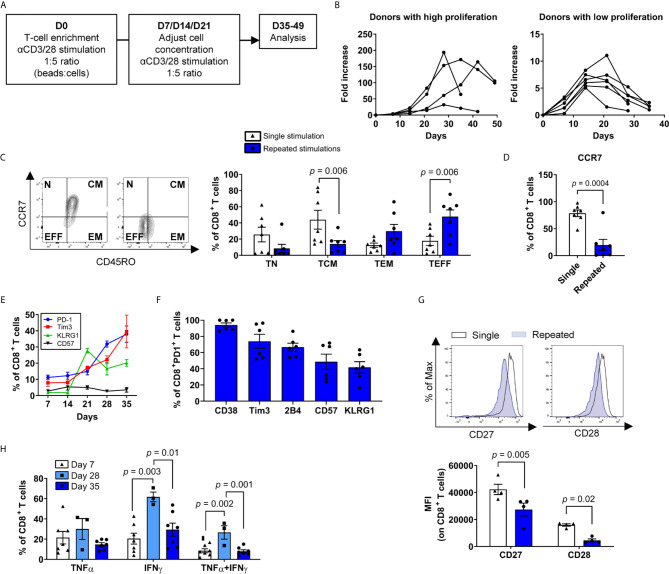
*Ex vivo* repeated polyclonal T-cell stimulations induce phenotypic and functional exhaustion. **(A)** Scheme of the general study design. **(B)** Growth curves of T-cell expansion from different donors. T cells were considered dysfunctional after one to two weeks of stagnant or negative growth (between 35 to 49 days for all donors) (n=10 independent donors). **(C)** Percentages of naïve, CD45RO^neg^, CCR7^pos^ (TN), central memory CD45RO^pos^, CCR7^pos^ (TCM), effector memory CD45RO^pos^, CCR7^neg^ (TEM) and effector CD45RO^neg^, CCR7^neg^ (TEFF) T cells after single or repeated T-cell stimulations (n=7 independent donors). **(D)** Percentage of CD8^+^ T cells expressing CCR7 after single or repeated T-cell stimulations (n=7 independent donors). **(E)** Immune checkpoints PD-1, Tim3, KLRG1 and CD57 expression among CD8^+^ T cells over time (n=6 independent donors). **(F)** Co-expression of exhaustion/dysfunction markers CD38, Tim3, 2B4 (CD244), CD57 and KLRG1 on PD-1-expressing CD8^+^ T cells (n=6 independent donors). **(G)** CD27 and CD28 expression on repeatedly stimulated CD8^+^ T cells as assessed by the mean fluorescence intensity (MFI) Representative plot (top) and compiled data (bottom) are shown (n=4 independent donors). **(H)** Cytokine secretion profile (IFNγ, TNFα) of cells following stimulation with PMA/ionomycin (n=3-7 independent donors). A two-tailed student t-test was used for **(C, D, G)** ANOVA with Tukey *post-hoc* testing was used in **(H)**. Data are represented as means ± SEM.

To define the molecular events leading to T-cell dysfunction following repeated stimulations, we exposed primary human T cells to anti-CD3/CD28 antibody-coated beads weekly in culture (**Figure 1A**). Despite substantial donor-dependent variation in the magnitude of total T-cell proliferation, we consistently observed a strong initial expansion phase, followed by a cessation of T-cell growth typically after 5 to 7 rounds of stimulation (**Figure 1B**). T cells were characterized after a single stimulation (day 7) and compared with cells harvested after two consecutive weeks of halted proliferation, occurring between 35-49 days. As anticipated, serial stimulations promoted the accumulation of more differentiated effector memory (TEM) and effector (TEFF) phenotype T cells relative to central memory (TCM) phenotype T cells in both CD4^+^ and CD8^+^ subtypes (**Figure 1C** and **Supplementary Figure 1**). We next focused on CD8^+^ T cells to compare our findings to a large body of previous data on CD8^+^ T-cell differentiation and exhaustion. We noted a marked decrease in CCR7 expression as well as co-expression of multiple inhibitory receptors as the cultures progressed over time (**Figures 1D–F**). In contrast, costimulatory molecules CD27 and CD28 expression decreased as expected for dysfunctional T cells (41) (**Figure 1G**). In addition, the percentage of cytokine-producing T cells declined between the week prior to culture termination (day 28) and the end of the culture (day 35) (**Figure 1H**). Thus, repeated *ex vivo* CD3/CD28 stimulations induce classical T-cell differentiation and exhaustion features.

### Repeated Stimulations Program an Exhaustion Transcriptome

**Figure 2:**
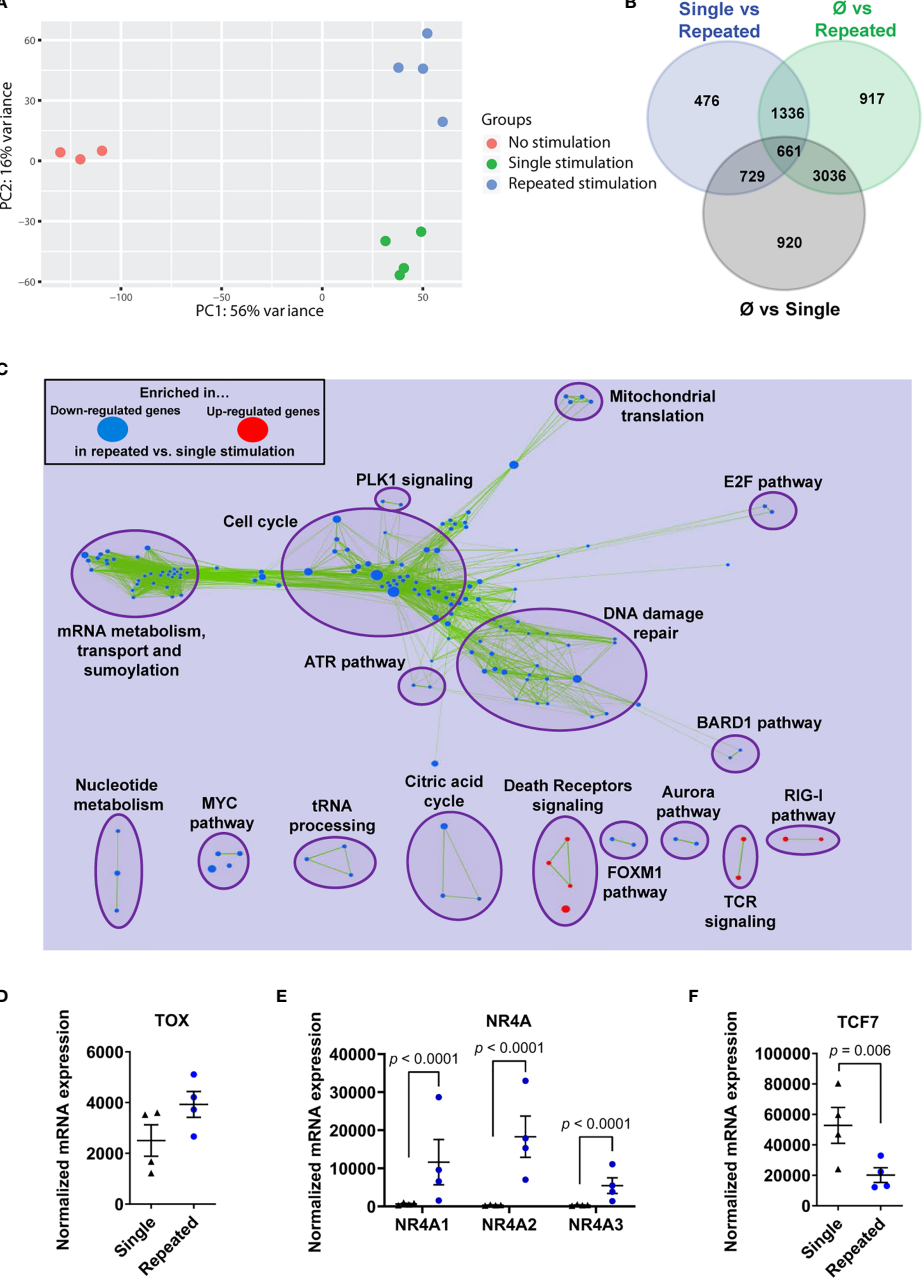
Repeated *ex vivo* stimulations program an exhaustion transcriptome. **(A)** Principal component analysis of non-stimulated, single stimulated and repeatedly stimulated CD8^+^ T cells. **(B)** Venn diagram of genes differentially regulated between groups (n=4 independent donors). **(C)** Gene-set enrichment analysis (GSEA) on RNA-seq gene transcript expression, loaded in Cytoscape (Enrichment Map plugin) and filtered for significance according to the p-value (0.001) and FDR (0.01) thresholds (n=4 independent donors). **(D)** RNA sequencing of enriched CD8^+^ T cells showing expression of *TOX* in repeatedly stimulated cells compared to single time stimulation (n=4 independent donors). **(E)** RNA sequencing of enriched CD8^+^ T cells showing expression of *NR4A* isoforms in repeatedly stimulated cells compared to single time stimulation (n=4 independent donors). **(F)** RNA sequencing of enriched CD8^+^ T cells showing expression of *TCF7* in repeatedly stimulated cells compared to single time stimulation (n=4 independent donors). Overlap between significant gene-sets, computed according to the Jaccard + overlap combined coefficient, was used for **(C)** A Wald test corrected for multiple testing using the Benjamini and Hochberg method was used for RNA-Seq data in **(D, F)**. Data are represented as means ± SEM.

We then proceeded to transcriptional profiling after a single stimulation (day 7) and repeatedly stimulated dysfunctional CD8^+^ T cells (day 35). Principal component analysis showed a clear segregation between time points and revealed a total of 476 gene transcripts differentially regulated between single and repeated stimulations (**Figures 2A, B**). By focusing on T-cell activation genes (GO:0042110), we readily identified the downregulation of TCM-associated (e.g. *CCR7* and *SELL*) and the upregulation of exhaustion-associated (e.g. *EOMES* and *HAVCR2*) mRNAs in repeatedly stimulated T cells relative to day 7 cells (**Supplementary Figure 2**). Integrated networks of gene set enrichment analysis pointed to several cell cycle-related pathways in dysfunctional T cells, but also the upregulation of genes in the T-cell receptor and death receptor signaling pathways (**Figure 2C**). The *TOX* mRNA expression, which encodes the master transcription factor regulating T-cell exhaustion fates, was slightly higher in repeatedly stimulated CD8^+^ T cells relative to day 7 T cells, which led to a significant increase of the three isoforms of its target transcription factors *NR4A* (42, 43) (**Figures 2D, E**). Along the same lines, dysfunctional CD8^+^ T cells lost the expression of *TCF7* mRNA (encoding TCF1), suggesting a state compatible with terminal exhaustion (42, 44) (**Figure 2F**). Collectively, these results confirm the development of T-cell exhaustion in repeatedly stimulated T cells *ex vivo*.

### Repeatedly Stimulated CD8^+^ T Cells Display a Strong Senescence-Associated Gene Signature

**Figure 3:**
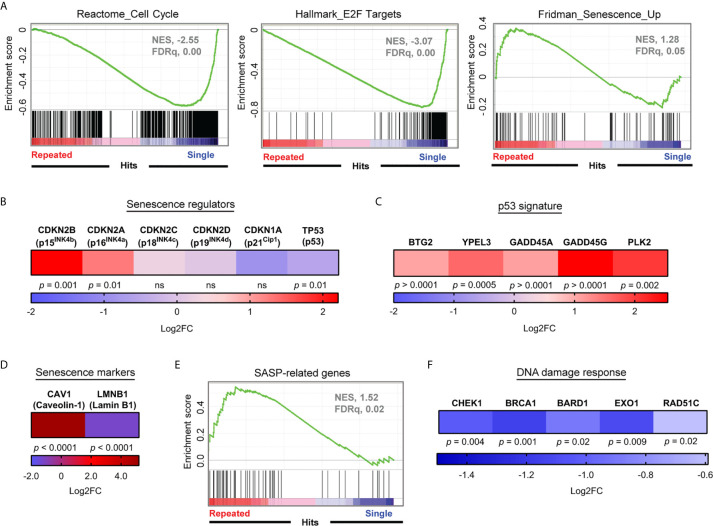
Repeatedly stimulated CD8^+^ T cells display a strong senescence-associated gene signature. **(A)** Gene Set Enrichment Analyses (Cell cycle, E2F targets and Senescence) performed on RNA-seq data normalized using the regularized logarithm (rlog) method provided in DESeq2 R package obtained from repeatedly stimulated compared to single stimulated enriched CD8^+^ T cells (n= 4 independent donors). **(B)** Differential expression of mRNA transcripts of key senescence regulators *CDKN2A* (p16^INK4a^), *CDKN2B* (p15^INK4b^), *CDKN2C* (p18^INK4c^), *CDKN2D* (p19^INK4d^), CDKN1A (p21^cip1^), and T53 (p53) (n= 4 independent donors). **(C)** Modulation of mRNA transcripts related to the senescence-associated p53 pathway in repeatedly stimulated cells compared to single stimulation (n=4 independent donors). **(D)** Caveolin-1 (*CAV1)* and lamin B1 (*LMNB1*) mRNA expression in repeatedly stimulated cells (n=4 independent donors). **(E)** Senescence-associated secretory phenotype (SASP) Gene Set Enrichment Analysis performed on RNA-seq data normalized using the regularized logarithm (rlog) method provided in DESeq2 R package obtained from repeatedly stimulated compared to single stimulated enriched CD8^+^ T cells (n= 4 independent donors). **(F)** Modulation of gene transcripts associated with the DNA Damage Response (n=4 independent donors). A Wald test corrected for multiple testing using the Benjamini and Hochberg method was used for RNA-Seq data in **(B–D, F)**.

Given that serial stimulations efficiently generated late-stage exhausted CD8^+^ T cells, we sought to identify the driver mechanisms responsible for their arrested expansion. Our transcriptome analysis revealed that prominent characteristics of repeatedly stimulated T cells were associated with cellular senescence such as the downregulation of genes involved in DNA repair processes (45), cell cycle and E2F target genes (37), as well as upregulated senescence-associated transcripts (46, 47) (**Figures 2C**, **3A** and **Supplementary Figure 2C**). More specifically, transcript levels of p16^INK4a^ and its related gene p15^INK4b^, central mediators of cellular senescence, were upregulated (**Figure 3B**). Due to the critical role of the p53 tumor suppressor pathway in cellular senescence, we also sought for evidence of contribution of this pathway upon repeated stimulations. Despite a decrease of *TP53* transcripts and the absence of induction of its classical downstream gene target *CDKN1A* in repeatedly stimulated CD8^+^ T cells, we observed the up-regulation of numerous p53 target genes, such as *BTG2*, *YPEL3*, *GADD45A/G*, and *PLK2*, which can contribute to cellular senescence (48) (**Figure 3C**). Other transcriptional features of cellular senescence were also found such as increased *CAV1* and the loss of *LMNB1* mRNAs (49, 50), coding for major structural proteins of the plasma membrane and nuclear lamina, respectively (**Figure 3D**). In addition, dysfunctional CD8^+^ T cells displayed elevated levels of transcripts related to the senescence-associated secretory phenotype (SASP) and a decreased expression of several genes required for DNA repair processes, which are features of cell senescence (45, 47, 51) (**Figures 3E, F**). Furthermore, when analyzing a previously published dataset (31) of circulating T cells from acute myeloid leukemia (AML) patients, a condition known to be associated with immune exhaustion (31, 52, 53), we noted high expression of transcripts encoding PD-1 along with p16^INK4a^, p21^Cip1^, and senescence-associated β-galactosidase (SA-β-Gal) (54) in T cells from patients relative to normal donors (**Supplementary Figure 3**). This adds to our findings inferring that exhaustion- and senescence-associated transcripts are co-regulated in dysfunctional T cells.

### Cellular Senescence Features Are Restricted to PD-1-Expressing T Cells

**Figure 4:**
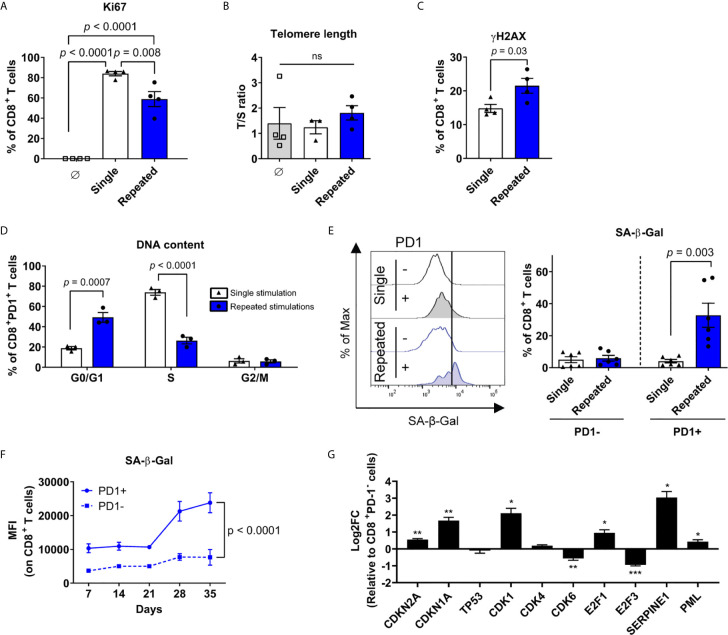
Cellular senescence features are restricted to PD-1-expressing T cells. **(A)** Proliferation marker (Ki67) expression on CD8^+^ T cells at the beginning of the culture, after a single, or repeated stimulations (n=4 independent donors). **(B)** Telomere length in non-activated CD8^+^ T cells, after single or repeated stimulations (n=4 independent donors). **(C)** Intracellular immunostaining of histone H2AX phosphorylation (yH2AX) after single or repeated stimulations (n=4 independent donors). **(D)** Proportions of CD8^+^PD1^+^ T cells in the G0/G1, S or G2/M cell cycle phase as assessed by DNA content following a single versus repeated stimulations (n=3 independent donors). **(E)** Enrichment of PD-1^+^ cells with increased senescence-associated-β-galactosidase (SA-β-Gal) activity in repeatedly stimulated cultures. Representative plot and compiled data are shown (n=6 independent donors). **(F)** Mean fluorescent intensity (MFI) of SA-β-Gal in PD-1^+^ compared to PD-1^-^ CD8^+^ T cells over time (n=3-6 independent donors). **(G)** mRNA expression (qPCR) of senescence-associated genes in sorted CD8^+^PD-1^+^ cells compared to control CD8^+^PD-1^-^ cells repeatedly stimulated (n=3 independent donors). ANOVA with Tukey *post-hoc* testing was used in **(A, B)** A two-tailed student t-test was used for **(C-E, G)** A mixed-effects model (REML) with Geisser-Greenhouse correction was performed for **(F)**. Data are represented as means ± SEM. *p < 0.05, **p < 0.01, ***p < 0.001, ns, non significant.

Consistent with growth arrest, the proportion of CD8^+^ T cells expressing the proliferation marker Ki67 was lower following repeated stimulations (**Figure 4A**). We next evaluated the telomere repeat copy number to single copy gene (T/S) ratio and confirmed that serially stimulated CD8^+^ T cells do not have critically short telomeres, hinting at a process independent of their replicative potential (**Figure 4B**). However, repeatedly stimulated CD8^+^ T cells accumulated histone H2AX phosphorylation indicative of DNA damage, another classical feature of cell senescence (55–57) (**Figure 4C**). We then interrogated whether exhaustion and cellular senescence were developing in the same T cells. Proliferation arrest was predominant among CD8^+^PD1^+^ T cells, which were found to be arrested in the G0/G1 phases of the cell cycle in greater proportion at the end of the culture than after a single stimulation (**Figure 4D**). We then compared PD-1-expressing and PD-1-negative CD8^+^ T cells. Remarkably, repeated stimulations led to an increase in SA-β-Gal activity following the third week (day 21), exclusively in PD-1^+^ cells (**Figures 4E, F**). Consistent with the data obtained with anti-CD3/CD28 coated beads, repeated stimulations with the HLA-A0201-restricted Epstein-Barr virus (EBV) epitope LMP2_426-434_ also led to a robust induction of SA-β-Gal in LMP2-specific PD-1-expressing T cells, confirming that the co-occurrence of cell senescence features and PD-1 expression is observed across several models (**Supplementary Figure 4**). When focussing on gene expression between repeatedly stimulated PD-1-expressing and PD-1-negative CD8^+^ T cells, both *CDKN2A* (p16^INK4a^) and *CDKN1A* (p21^Cip1^) were upregulated in the PD-1^+^ fraction, without any change in *TP53* (p53) expression (**Figure 4G**). Moreover, cyclin-dependent kinase 6 (*CDK6*) and *E2F3* transcription factor were negatively impacted in PD-1-expressing cells. These latter are both implicated in the progression into the S-phase of the cell cycle and mainly controlled by p16^INK4a^ (58, 59). Other senescence-associated gene transcripts, such as *SERPINE1* and *PML*, were also increased in PD-1^+^ T cells (48, 60–62) (**Figure 4G**). Altogether, these results show that transcriptomic, phenotypic and functional features of cellular senescence are restricted to repeatedly stimulated PD-1-expressing exhausted CD8^+^ T cells.

### Exhausted Antigen-Specific T Cells Exhibit Senescence-Associated Features *In Vivo*

**Figure 5:**
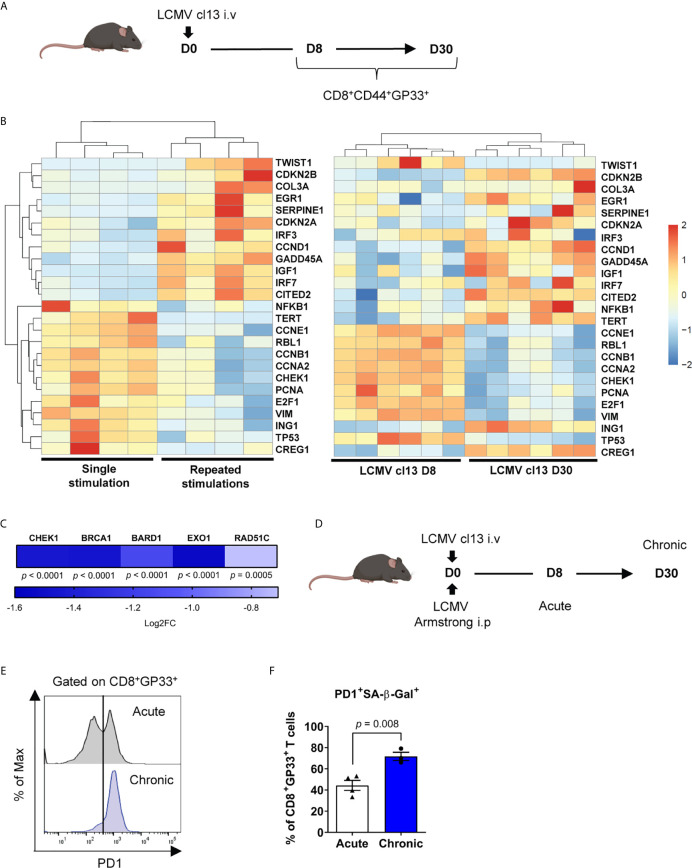
Exhausted antigen-specific T cells exhibit senescence-associated features *in vivo*. **(A)** Scheme of LCMV clone 13 (cl13) mouse infection. Mice were infected intravenously with LCMV and spleens were collected at day 8 and 30 post-infection. **(B)** RNA-seq of CD8^+^CD44^+^GP33^+^ cells showing senescence-associated mRNA profile from the chronic LCMV cl13 mouse model compared to the top 25 most differentially regulated senescence-associated genes in the human CD3/CD28 repeated stimulation model (n=6 mice). **(C)** Modulation in gene transcripts from RNA-Seq associated with the DNA Damage Response at day 8 and day 30 of chronic infection (LCMV clone 13) (n=6 mice). **(D)** Scheme of chronic LCMV cl13 compared to acute LCMV Armstrong mouse infection. **(E)** Representative plot of PD-1 expression among GP33-specific cells from acute (LCMV Armstrong day 8) and chronic infection (LCMV clone 13 day 30) (n=7-8 mice). **(F)** PD-1^+^ cells with increased SA-β-Gal activity in GP33-specific T cells from acute compared to chronic infection (n=3-4 mice; representative of 2 independent experiments). A Wald test corrected for multiple testing using the Benjamini and Hochberg method was used for RNA-Seq data in **(C)**. A two-tailed student t-test was performed in **(F)**. Data are represented as means ± SEM.

Long term culture can impose a selection pressure on *ex vivo* expanded T cells and bias their differentiation state (63). We thus used the well-described mouse T-cell exhaustion model of lymphocytic choriomeningitis virus (LCMV) infection to determine whether our findings extended to this *in vivo* model. We first compared virus-specific CD8^+^ T cells in early and late chronic LCMV clone 13 (cl13) infection and noted that exhausted GP33-specific CD8^+^ T cells share a similar senescence-associated mRNA expression pattern when compared to dysfunctional human CD3/CD28 stimulated CD8^+^ T cells (**Figures 5A, B**). Indeed, *CDKN2A* and *CDKN2B* along with *SERPINE1* were likewise upregulated, while cyclins (e.g. *CCNE1*, *CCNB1* and CCNA1) were downregulated (**Figure 5B**). In addition, these exhausted T cells showed a downregulation of gene transcripts required for DNA damage repair processes as seen for human dysfunctional T cells (**Figure 5C**). Moreover, chronic infection promoted the accumulation of a higher proportion of GP33-specific cells expressing PD-1 and increased SA-β-Gal activity as compared to an acute infection (**Figures 5D–F**), confirming that CD8^+^ T-cell dysfunction is associated with features of senescence in exhausted PD-1^+^ cells *in vivo*.

### Inhibition of p38MAPK Limits DNA Damage and T-Cell Senescence

**Figure 6:**
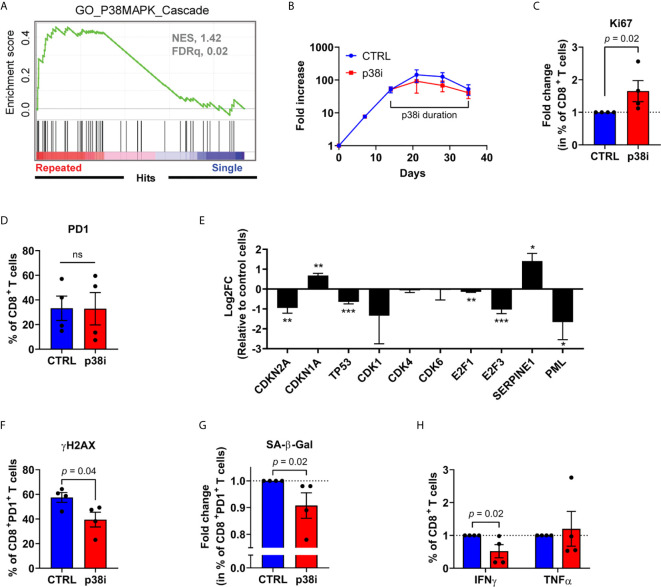
Inhibition of p38MAPK limits DNA damage and T-cell senescence. **(A)** p38MAPK Cascade Gene Set Enrichment Analysis performed on RNA-seq data normalized using the regularized logarithm (rlog) method provided in DESeq2 R package obtained from repeatedly stimulated compared to single stimulated enriched CD8^+^ T cells (n= 4 independent donors). **(B)** Expansion of repeatedly stimulated T cells treated or not with 500 nM of BIRB796 (p38i) from day 14 to the end of the culture (n=4 independent donors). **(C)** Fold change in percentage of repeatedly stimulated CD8^+^ expressing the proliferation marker Ki67 from p38i-treated compared to control culture (n=4 independent donors). **(D)** PD-1 expression on CD8^+^ T cells from p38i-treated compared to control culture (n=4 independent donors). **(E)** mRNA expression (qPCR) of senescence-associated genes in sorted repeatedly stimulated CD8^+^PD-1^+^ cells treated or not (control cells) with p38i (n=3 independent donors). **(F)** Intracellular immunostaining of histone H2AX phosphorylation (yH2AX) on repeatedly stimulated CD8^+^PD-1^+^ cells treated or not with p38i (n=4 independent donors). **(G)** Fold change in percentage of repeatedly stimulated CD8^+^PD-1^+^ cells with increased SA-β-Gal activity treated or not with p38i (n=4 independent donors). **(H)** Fold change in percentage of cytokine-secreting (IFNγ, TNFα) repeatedly stimulated CD8^+^ T cells treated or not with p38i (n=4 independent donors). A two-tailed student t-test was performed in **(D–F)**. A two-tailed Mann-Whitney test was performed for **(C, G, H)**. Data are represented as means ± SEM. *p < 0.05, **p < 0.01, ***p < 0.001, ns, non significant.

The p38MAPK pathway is closely related to cellular senescence development, and is active in terminally differentiated T cells (27, 28). Likewise, repeatedly stimulated CD8^+^ T cells *ex vivo* expressed a transcriptomic signature linked to the p38MAPK signaling cascade (**Figure 6A**). Given that short term p38MAPK inhibition with 500 nM of the p38MAPK inhibitor BIRB796 *ex vivo* has been previously associated with improved T-cell function (27, 28), we sought to evaluate whether it may also reverse established cellular senescence features in serially activated dysfunctional T cells. To this end, we added BIRB796 (hereafter referred to as p38i) to the cell cultures following the third CD3/CD28 stimulation (**Figure 6B**), corresponding to the timing of increased SA-β-Gal activity detection in PD-1^+^ cells (**Figure 4F**). While p38i did not impact overall T-cell recovery by the end of the culture, nor the percentage of PD-1-expressing CD8^+^ T cells, it led to a greater accumulation of Ki67^+^ cells (**Figures 6B–D**). When focussing specifically on PD-1-expressing CD8^+^ T cells, we noted that p38i reduced the expression of *CDKN2A* (p16^INK4a^), *TP53* and *PML* (**Figure 6E**). It also correlated with a decreased percentage of PD-1-expressing cells displaying DNA damage and with SA-β-Gal activity (**Figures 6F, G**). It is noteworthy to point out that despite the mitigation of senescence features, this specific dose of p38 inhibitor did not improve, but rather decreased the IFNγ-secreting potential of T cells (**Figure 6H**). Thus, the p38MAPK signaling pathway is implicated in T-cell dysfunction and p16^INK4a^ upregulation. However, despite the positive effects of p38i on certain senescence-associated features, the pharmacological blockade of p38MAPK after several rounds of stimulation failed to restore T-cell functionality.

### Inhibition of p16^INK4a^ Expression Reinvigorates Dysfunctional T Cells

**Figure 7:**
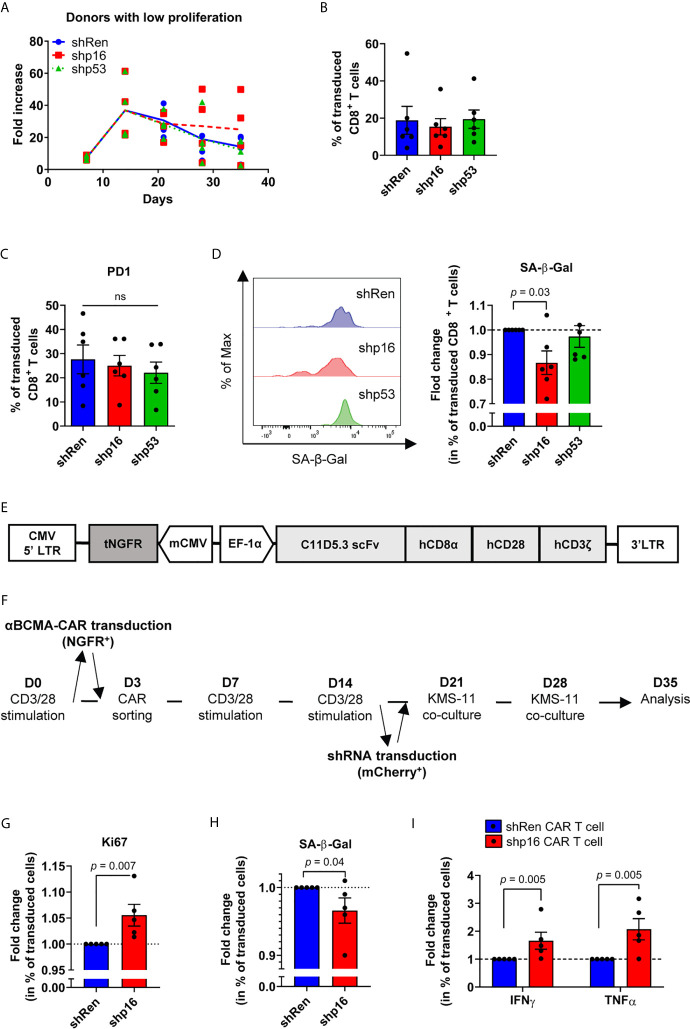
Modulation of p16^INK4a^ expression reinvigorates dysfunctional T cells. **(A)** Expansion of repeatedly stimulated T cells from donors with a low proliferation profile transduced with p16^INK4a^ (*CDKN2A*), *TP53* (p53) or non-targeting (Ren; renilla) shRNA 24 hours after the third stimulation (n=6 independent donors). **(B)** Percentage of transduced CD8^+^ T cells by the end of the culture (n=6 independent donors). **(C)** PD-1 expression on transduced CD8^+^ T cells (n=6 independent donors). **(D)** Fold change in percentage of transduced CD8^+^ T cells with increased SA-β-Gal activity. Representative plot and compiled data are shown (n=6 independent donors). **(E)** Scheme of the BCMA-specific CAR vector containing a truncated NGFR (tNGFR) as a transduction control under the control of a mCMV promoter and the human BCMA-CD3ζ-CD28-CD8α complex driven by the EF-1α promoter. **(F)** Scheme of BCMA-specific CAR T cell generation transduced with p16^INK4a^ (*CDKN2A*) or non-targeting shRNA 24 hours after the third CD3/CD28 stimulation, then co-cultured for two consecutive weeks with BCMA-expressing human multiple myeloma cells. **(G)** Fold change in percentage shRNA-transduced BCMA-specific CAR T cells expressing the proliferation marker Ki67 (n=5 independent donors). **(H)** Fold change in percentage of shRNA-transduced BCMA-specific CAR T cells with increased SA-β-Gal activity (n=5 independent donors). **(I)** Fold change in percentage of cytokine-secreting (IFNγ, TNFα) shRNA-transduced BCMA-specific CAR T cells (n=5 independent donors). ANOVA with Tukey *post-hoc* testing was used in **(C)** A two-tailed Mann-Whitney test compared to control condition was used for **(D, G, H, I)** Data are represented as means ± SEM. ns, non significant.

Since pharmacologic p38i can downregulate both p16^INK4a^ and p53 transcripts in PD-1-expressing exhausted CD8^+^ T cells, we sought to gain more insights into which pathway downstream of p38MAPK is responsible for conferring senescence features to dysfunctional T cells. We thus performed knockdown of p16^INK4a^ and p53 with validated shRNA vectors (37, 38). Knockdown performed following the third CD3/CD28 stimulation did not significantly affect global expansion nor PD-1 expression (as for p38i) in repeatedly stimulated cultures (**Figures 7A–C**). However, p16^INK4a^, but not p53 targeting, successfully limited the development of CD8^+^ T cells with increased SA-β-Gal activity (**Figure 7D**). We next used a clinically relevant approach to investigate cell fate in context of initial CD3/CD28 stimulation followed by serial antigenic encounters. We generated B-cell maturation antigen (BCMA)-specific CAR T cells (40) that were activated and expanded for three weeks with anti-CD3/CD28 beads and subsequently transduced with p16^INK4a^ *-*targeting or non-targeting shRNAs (**Figures 7E, F**). While exposure to repetitive stimulations with BCMA-expressing human KMS-11 multiple myeloma cells (64) led to the expression of inhibitory receptors such as PD-1 and KLRG1 (**Supplementary Figure 5**), p16^INK4a^ knockdown increased the fraction of Ki67^+^ cells and restricted the development of SA-β-Gal-expressing CAR T cells (**Figures 7G, H**). In contrast with data obtained with p38i, directly modulating p16^INK4a^ expression increased the proportion of cytokine-secreting T cells in this setting (**Figure 7I**). Thus, our results in *ex vivo* repeatedly bead-stimulated T cells dovetail with findings in several other experimental systems and establish p16^INK4a^ as a key mediator of activation-induced T-cell senescence and dysfunctionality.

## Discussion

The loss of T-cell function following repeated stimulations is a major limitation for the control of chronic viral infections and cancer. Modern cancer immunotherapy hinges on the prevention or reversal of T-cell dysfunction, either through immune checkpoint blockade or adoptive cell therapy. In the latter situation, *ex vivo* manipulations prior to T-cell infusion, as well as repeated stimulations after adoptive transfer *in vivo*, often induce T-cell dysfunction, which severely impedes the therapeutic potential of this approach. However, the processes articulating the development and maintenance of T-cell dysfunction remain elusive (65).

Among T-cell dysfunctional states, exhaustion occurs when the native T-cell or synthetic (i.e. CAR) receptor is strongly and repeatedly engaged, in presence of various inflammatory cytokines. Exhausted T cells then progressively show diminished proliferative and functional capacities while expressing high levels of immune checkpoints such as PD-1 (66, 67). In line with this, we found that repeatedly stimulated T cells *ex vivo* displayed a gradual accumulation of CD8^+^ T cells co-expressing multiple inhibitory receptors along with a reduced cytokine-secretion capacity and a marked growth arrest. In addition, our transcriptional profile showed the co-occurrence of a T-cell exhaustion and senescence program among repeatedly stimulated CD8^+^ T cells. We further established the strong relationship between PD-1 expression and senescence features in individual T cells in several experimental systems in human and mouse. Whether such co-occurrence depends on a co-regulation or distinct mechanisms acting in parallel remains unclear. However, our data argue against the notion that exhaustion and senescence are mutually exclusive pathophysiologically.

We extend these findings showing that growth arrest in *ex vivo* stimulated T cells is not related to abnormal telomere length, but rather associated with activation-induced genotoxic stress and DNA damage. DNA double strand breaks can initiate p38MAPK signaling cascade. When phosphorylated, p38 mediates the activation of several transcription factors and inflammatory cytokines (68). It has also been reported that p38MAPK can induce p16^INK4a^ transcription as well as activate p53 following direct phosphorylation on Ser33 to mediate cell cycle arrest (12, 13, 69). The inhibition of p38MAPK in the context of a brief *ex vivo* restimulation of terminally differentiated and γH2AX-expressing T cells was shown to reverse senescence features (27, 28). Given the pleiotropic effects of p38MAPK signaling, it was unclear whether p38i would reverse T-cell senescence features and promote functionality in the context of ongoing stimulations once senescence features become conspicuous. In repeatedly stimulated T cells, inhibiting p38MAPK signaling after the third T-cell stimulation could limit the development of senescence features by reducing the expression of p16^INK4a^, limiting DNA damage and SA-β-Gal activity in PD-1-expressing cells. Although it also reduced the expression of p53, p38i treatment promoted the increase of p21^Cip1^, suggesting that an alternative senescence mechanism from the p53/p21^Cip1^ axis may still take place. Consistent with previous reports showing a correlation between p38MAPK inhibition and decreased IFNγ expression (70), exposing T cells to p38i during repeated TCR stimulations failed to restore T-cell functionality in terms of pro-inflammatory cytokine secretion despite improvements in senescence-associated features. Although we cannot exclude the possibility that modulating the dosage or method of p38MAPK inhibition may result in different outcomes, p38i in the context of serially activated T cells could alleviate DNA damage and senescence features but this did not result in functional improvements, suggesting that timing of p38MAPK modulation in the context of immunotherapy must be carefully planned.

Our data indicate that T-cell cellular senescence in the context of chronic activation is maintained by p16^INK4a^ regulation as *CDKN2A* silencing after the third stimulation could attenuate senescence features. We further show that CAR T cells serially stimulated with cancer cells bearing the targeted antigens develop dysfunctionality in terms of dual expression of exhaustion and senescence features, which can also reflect the fate of adoptively transferred cells. Whether tonic signaling associated with CAR T-cell exhaustion (71, 72) also results in direct induction of senescence features is currently unknown but may represent a factor impeding CAR T-cell efficacy. Similar to our CD3/CD28 stimulation model, the targeting of p16^INK4a^ downstream of p38MAPK likewise reversed senescence features, yet it could also improve cytokine secretion capacity among serially activated CAR T cells. This enhancement in cytokine secretion was not accompanied by decreased PD-1 expression or an increased expansion, which suggest the contribution of other growth regulatory mechanisms. Despite p16^INK4a^ being predominantly activated, we cannot rule out the potential contribution of the p53/p21^Cip1^ pathway. Indeed, we observed elevated mRNA levels of p21^Cip1^ in PD-1-expressing dysfunctional CD8^+^ T cells, arguing in favor of its potential role in early senescence initiation (11, 12, 73). This may explain why certain senescence features are not reversed following p16^INK4a^ or p38MAPK targeting. This is reassuring as interfering with p16^INK4a^ expression in senescent cells raises the concern of inducing malignant transformation. It was previously shown that p16^INK4a^-deficient mouse T cells do not undergo cancerization (74), perhaps through the induction of such compensatory mechanisms. Finally, in the context where senescent cells can exhibit signs of resistance to checkpoint blockade (75, 76), p16^INK4a^ may be considered as an actionable target to alleviate senescence features in repeatedly stimulated PD-1-expressing T cells while also preserving cytokine-secretion capacity for therapeutic purposes.

## Data Availability Statement

The datasets presented in this study can be found in online repositories. The names of the repository/repositories and accession number(s) can be found below: https://www.ncbi.nlm.nih.gov/geo/, GSE132727; https://www.ncbi.nlm.nih.gov/geo/, GSE132989.

## Ethics Statement

The studies involving human participants were reviewed and approved by Hôpital Maisonneuve-Rosemont research ethics authorities. The patients/participants provided their written informed consent to participate in this study. The animal study was reviewed and approved by Hôpital Maisonneuve-Rosemont Animal Care Committee in accordance with the Canadian Council on Animal Care guidelines.

## Author Contributions

VJ and J-SD designed the study. VJ performed experiments and analyzed the data. MN did the shRNA constructions. M-ÈL performed LCMV mouse infections. DDS and SB did the mouse RNA-Seq. LD did the EBV-specific T-cell cultures. CM provided technical help. CC and SL helped with conceptualization. VJ and J-SD wrote the manuscript. NL, HM, FM, and J-SD supervised the study. All authors contributed to the article and approved the submitted version.

## Funding

This work was supported by the Richard and Edith Strauss Foundation operating grant (held by J-SD) and Canadian Institute for Health Research operating grants (PJT-152988 to NL; PJT-156133 to J-SD, HM, and FM).

## Conflict of Interest

The authors declare that the research was conducted in the absence of any commercial or financial relationships that could be construed as a potential conflict of interest.

## Publisher’s Note

All claims expressed in this article are solely those of the authors and do not necessarily represent those of their affiliated organizations, or those of the publisher, the editors and the reviewers. Any product that may be evaluated in this article, or claim that may be made by its manufacturer, is not guaranteed or endorsed by the publisher.
