# Supplementary material for: p16INK4a Regulates Cellular Senescence in PD-1-Expressing Human T Cells
Source: Front Immunol. 2021 Aug 9;12:698565. doi: 10.3389/fimmu.2021.698565 (PMC8381277; doi:10.3389/fimmu.2021.698565)
Supplement: Supplementary file 1 [file DataSheet_1.docx]

Supplementary Material

# Supplementary Figures

**
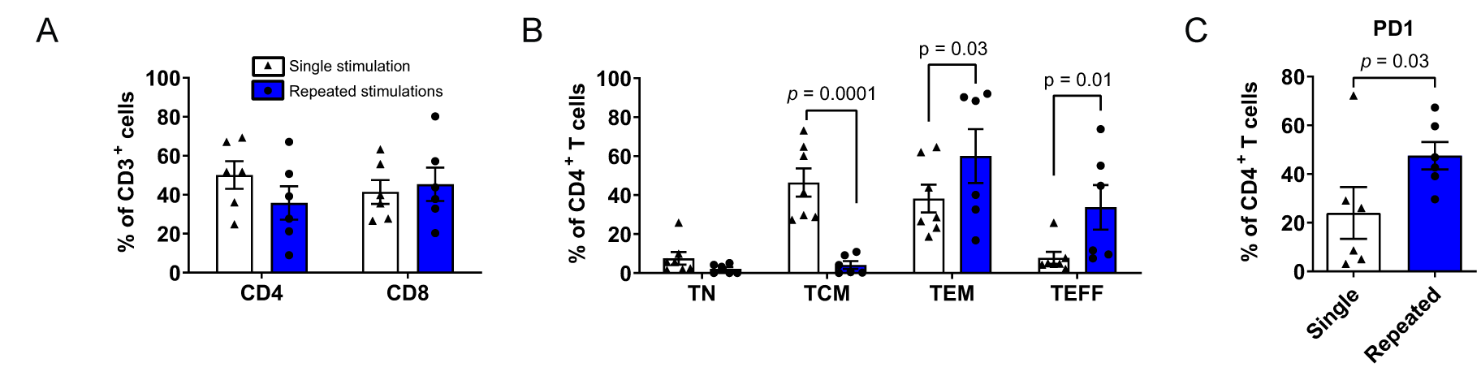
**

**Supplementary Figure 1.** **Repeated stimulations induce CD4^+^ T-cell differentiation and exhaustion.** Enriched human T cells were activated with a single stimulation or were stimulated weekly with anti-CD3/CD28 coated beads until two consecutive weeks of stagnant or negative growth (repeated). **(A)** CD4^+^ and CD8^+^ T-cell proportions after repeated stimulations (n=6 independent donors). **(B)** Repeated stimulations favored the accumulation of effector (TEFF) and effector memory (TEM) CD4^+^ T cells at the expense of central memory (TCM) T cells. TN; naïve T cells (n=7 independent donors). **(C)** Expression of the checkpoint inhibitor PD-1 following a single or repeated stimulations in CD4^+^ T cells (n=6 independent donors). A two-tailed student t-test was used for B and C. Data are represented as means ± SEM.


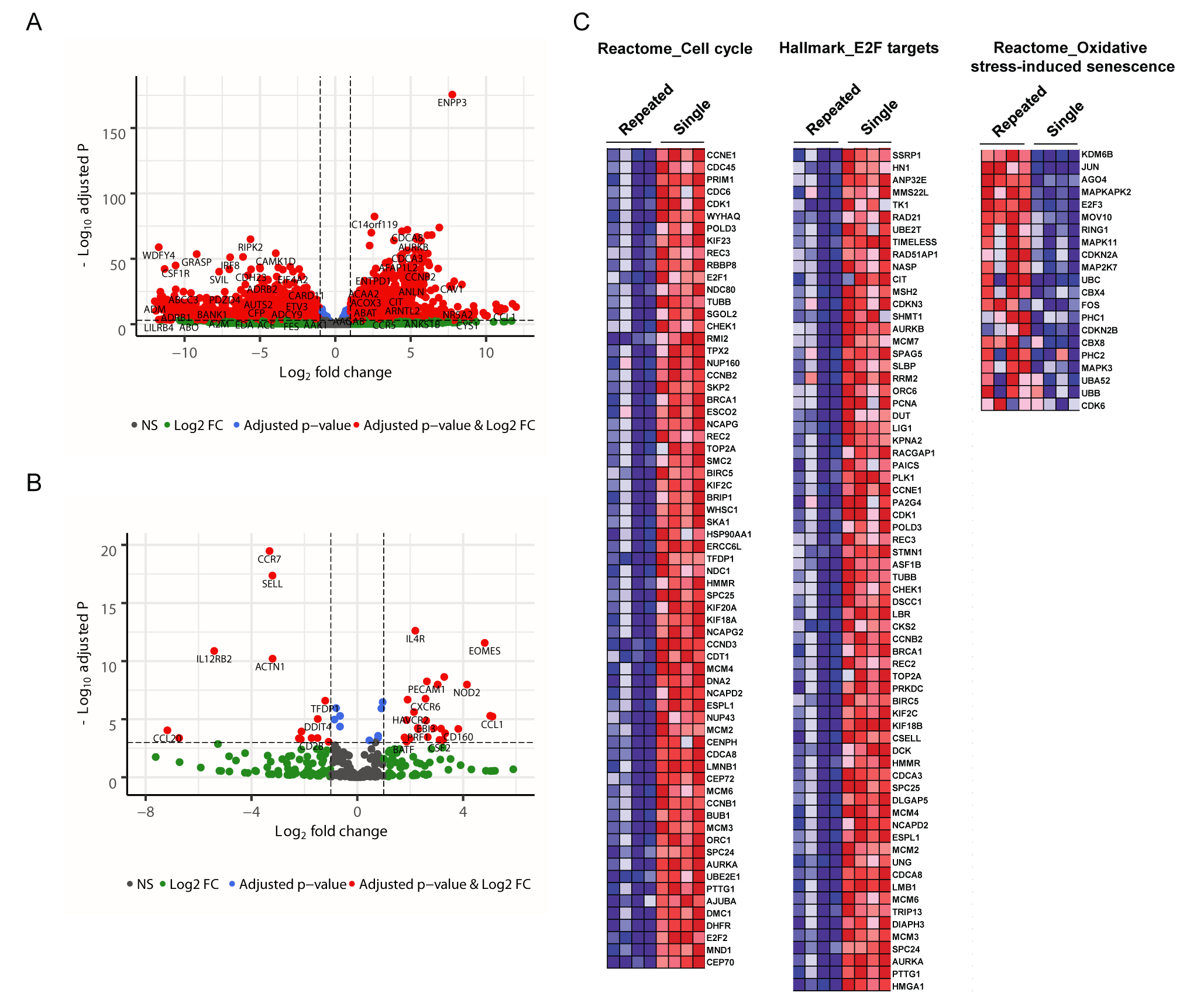


**Supplementary Figure 2. Global gene expression analysis of CD8^+^ T cells repeatedly stimulated compared to one stimulation alone.** CD8^+^ T cells were enriched from weekly anti-CD3/CD28 coated beads stimulated T cells and prepared for RNA sequencing at day 0, following a single stimulation (day 7) and after two weeks of stagnant or negative growth (repeated stimulations). **(A)** Volcano plot of all significantly differentially regulated transcripts (red; ≥ 2 fold change (FC) ; padj < 0.01) in dysfunctional CD8^+^ T cells (n=4 independent donors). **(B)** Volcano plot of T-cell activation gene transcripts in repeated versus single stimulated CD8^+^ T cells (n=4 independent donors). **(C)** Top differentially regulated genes within the Reactor_Cell cycle, Hallmark_E2F targets and Reactome_Oxidative stress-induced senescence gene collections (blue, downregulated; red, upregulated; n=4 independent donors).


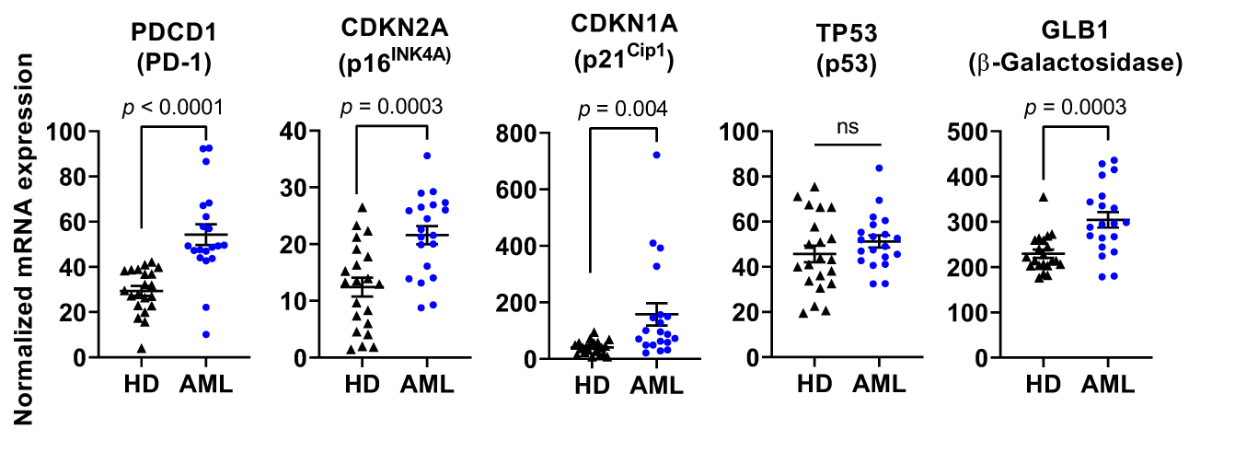


**Supplementary Figure 3. Circulating exhausted T cells from AML patients display a senescence-associated gene signature.** Differential expression of mRNA transcripts of *PDCD1* (PD-1), *CDKN2A* (p16^INK4a^), *CDKN1A* (p21^Cip1^), and *GLB1* (β-Galactosidase) in circulating T cells from healthy donors (HD) compared to acute myeloid leukemia (AML) patients (pooled samples from AML CD4^+^ T cells (n=10), AML CD8^+^ T cells (n=10), HD CD4^+^ T cells (n=10), HD CD8^+^ T cells (n=11)). A Wald test corrected for multiple testing using the Benjamini and Hochberg method was used for statistical significance. Data are represented as means ± SEM.


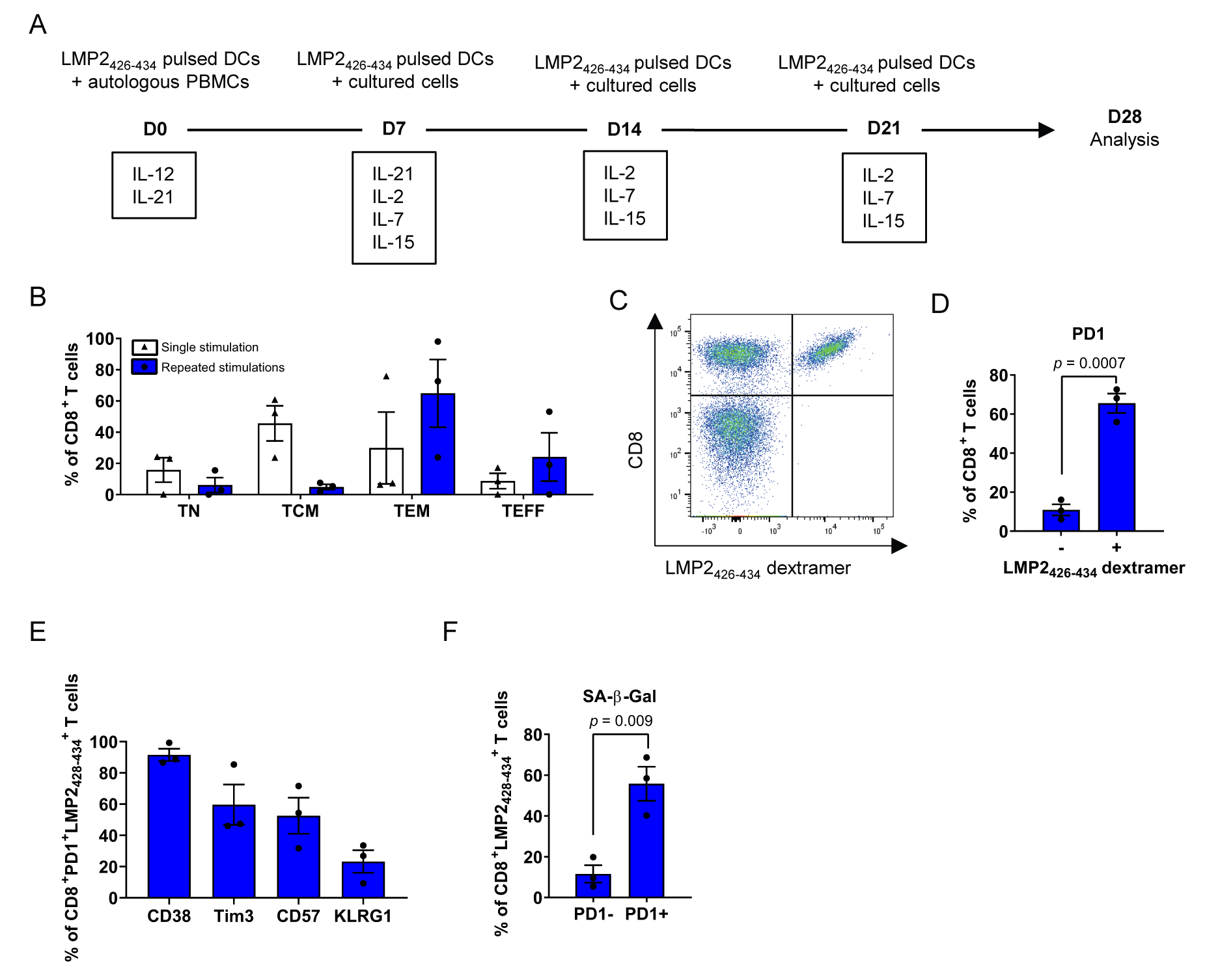


**Supplementary Figure 4. Epstein-Barr Virus (EBV)-specific human T cells repeatedly stimulated with peptide-pulsed dendritic cells display senescence markers associated with PD-1 expression.** Human monocyte-derived dendritic cells were generated, matured and pulsed with the EBV-LMP2_426-434_ peptide prior to co-culture with T cells. **(A)** Schematic of protocol used for antigen-specific T-cell lines generation. **(B)** Differentiation status of CD8^+^ cells after 3 to 4 weekly stimulations. (TN; naïve, TCM; central memory, TEM; effector memory, TEFF; effector T cells – n=3 independent donors). **(C)** Identification of LMP2-specific T cells using fluorescent HLA-peptide dextramer staining (one representative of 3 independent cultures). **(D)** PD-1 expression between the dextramer-positive and negative CD8^+^ T-cell fractions within the culture (n=3 independent donors). **(E)** Accumulation of LMP2-specific cells co-expressing PD-1 and other exhaustion/dysfunction markers such as CD38, Tim3, CD57 and KLRG1 (n=3 independent donors). **(F)** Expression of SA-β-Galactosidase restricted to PD-1-expressing LMP2_426-434_-specific T cells following multiple stimulations (n=3 independent donors). A two-tailed student t-test was used for D and E. Data are represented as means ± SEM.


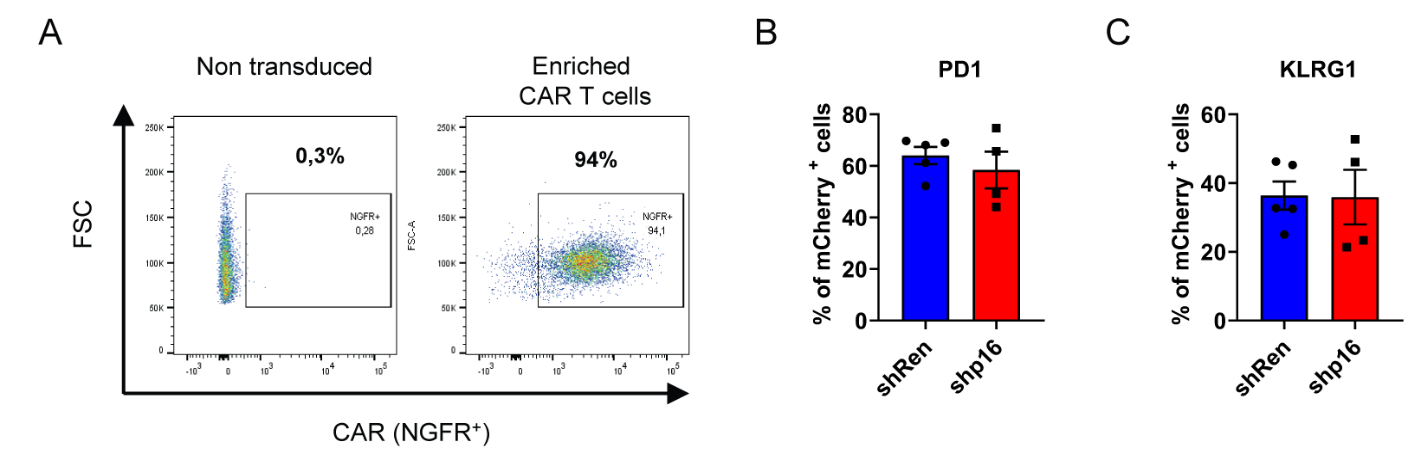


**Supplementary Figure 5. Repeated antigen encounters induce dysfunctionality in CAR T cells.** (**A**) Representative plot of CAR T-cell population after sorting. (**B**) PD-1 and (**D**) KLRG1 expression on shRNA-transduced BCMA-specific CAR T cells co-cultured for two consecutive weeks with BCMA-expressing human multiple myeloma cells (n=4 independent donors). Data are represented as means ± SEM.
